# A Complex Digital Health Intervention to Support People With HIV: Organizational Readiness Survey Study and Preimplementation Planning for a Hybrid Effectiveness-Implementation Study

**DOI:** 10.2196/76327

**Published:** 2026-01-21

**Authors:** Jacqueline Hodges, Wendy Cohn, Amanda Castel, Tabor Flickinger, Ava Lena Waldman, Michelle Hilgart, Olivia Kirby, Sylvia Caldwell, Karen Ingersoll

**Affiliations:** 1 Division of Infectious Diseases, Department of Medicine, Duke University, 315 Trent Drive, Durham, NC, 27710, United States, 1 919 681 1309, 1 434 404 4578; 2 Department of Public Health Sciences, University of Virginia, Charlottesville, VA, United States; 3 Milken Institute School of Public Health, George Washington University, Washington, DC, United States; 4 General, Geriatric & Palliative Medicine, Department of Medicine, University of Virginia, Charlottesville, VA, United States; 5 Division of Infectious Diseases and International Health, Department of Medicine, University of Virginia, Charlottesville, VA, United States; 6 Department of Psychiatry and Neurobehavioral Sciences, University of Virginia, Charlottesville, VA, United States

**Keywords:** mobile health, digital health, HIV, RE-AIM, CFIR, hybrid effectiveness-implementation study, implementation science, Reach Effectiveness Adoption Implementation Maintenance, Consolidated Framework for Implementation Research

## Abstract

**Background:**

Evaluating implementation of digital health interventions (DHIs) in practice settings is complex, involving diverse users and multistep processes. Proactive planning can ensure implementation determinants and outcomes are captured for hybrid studies, but operational guidance for designing or planning hybrid DHI studies is limited.

**Objective:**

This study aimed to proactively define, prioritize, and operationalize measurement of implementation outcomes and determinants for a DHI hybrid effectiveness-implementation trial. We describe unique advantages and limitations of planning the trial implementation evaluation among a large-scale cohort study population and share results of a pretrial organizational readiness assessment.

**Methods:**

We planned a cluster-randomized, type II hybrid effectiveness-implementation trial testing *PositiveLinks*, a smartphone app for HIV care, compared to usual care (n=6 sites per arm), among HIV outpatient sites in the DC Cohort Longitudinal HIV Study in Washington, DC. We (1) defined components of the DHI and associated implementation strategy; (2) selected implementation science frameworks to accomplish evaluation aims; (3) mapped framework dimensions, domains, and constructs to implementation strategy steps; (4) modified or created instruments to collect data for implementation outcome measures and determinants; and (5) developed a compatible implementation science data collection and management plan. Provider baseline surveys administered at intervention sites probed usage of digital tools and assessed provider readiness for implementation with the Organizational Readiness to Implement Change tool.

**Results:**

We specified DHI and implementation strategy toward planning measurement of DHI and broader program reach and adoption. Mapping of implementation strategy steps to the Reach Effectiveness Adoption Implementation Maintenance framework prompted considerations for how to capture understudied aspects of each dimension: denominators and demographic representativeness within reach or adoption, and provider or organization-level adaptations, dose, and fidelity within the implementation dimension. Our process also prompted the creation of tools to obtain detailed determinants across domains and constructs of the Consolidated Framework for Implementation Research within a large sample at multiple time points. Some aspects of real-world *PositiveLinks* implementation were not reflected within the planned hybrid trial (eg, research assistants selected as de facto site implementation leads) or were modified to preserve internal validity of effectiveness measurement (eg, “Community of Practice”). Providers and research assistants (n=17) at intervention sites self-reported high baseline use of digital tools to communicate with patients. Readiness assessment revealed high median (48, IQR 45‐54) total Organizational Readiness to Implement Change scores, with research assistants scoring higher than physicians (52.5, IQR 44-55 vs 48.0, IQR 46-49).

**Conclusions:**

Key takeaways, challenges, and opportunities arose in planning the implementation evaluation within a hybrid DHI trial among a cohort population. Prospective trial planning must balance generalizability of implementation processes to “real world” conditions with rigorous procedures to measure intervention effectiveness. Rapid, scalable tools require further study to enable evaluations within large multisite hybrid studies.

## Introduction

Digital health interventions (DHIs) including web-based and mobile health (mHealth) interventions can improve clinical outcomes for chronic health conditions. DHIs apply behavior change theories with variable mechanisms of action, such as enhanced motivation, self-management, and peer support. Munoz et al [1] and Hermes et al [2] describe the spectrum of behavioral intervention technologies ranging from adjunctive tools embedded within clinic-based care to support provider tasks, to fully-automated direct-to-consumer technologies for patient self-management. Implementation outcomes or the ‘who,’ ‘how,’ ‘how much,’ and ‘how well’ needed to implement DHIs [3] can vary widely as a result. Clinic-embedded DHIs may require significant engagement by multiple providers and staff. Additionally, DHIs with multiple features can generate large amounts of backend paradata related to usage that are important for quantifying implementation reach and adoption. Capturing DHI implementation outcomes and determinants can thus be complicated. Curran et al [4] describe 3 designs of hybrid effectiveness-implementation studies. However, the literature provides little operational guidance or shared procedural knowledge on planning hybrid studies to capture implementation outcomes and identify salient implementation determinants for complex multifeature multiuser DHIs.

We reviewed several comprehensive efforts to recharacterize or adapt broader health service implementation science frameworks for the study of DHIs. Recent examples related to evaluation frameworks, which evaluate implementation outcomes, include (1) a workshop conducted by the Dissemination and Implementation Core of the Center for Technology and Behavioral Health at Dartmouth College [5], (2) Hermes et al’s [2] recharacterization of Proctor’s outcomes for implementation research for technology-based behavioral interventions, and (3) De la Vega et al’s [6] post hoc app of this recategorized framework against Glasgow’s Reach Effectiveness Adoption Implementation Maintenance (RE-AIM) framework [7,8]. Determinant frameworks answer the question: ‘Why was the intervention/practice/innovation implemented or not implemented?’ Several frameworks applied to DHI implementation research include the Consolidated Framework for Implementation Research (CFIR) [9-12], the Theoretical Domains Framework [13], Promoting Action on Research Implementation in Health Services [14], and others. Application of these frameworks for DHI trials is often done post hoc rather than proactively, and practical guidance on incorporating frameworks into DHI hybrid trial planning is limited, despite their importance in revealing why implementation of DHIs tested within real-world settings did or did not meet expectations.

The *PositiveLinks* platform is a clinic-embedded multifeature smartphone app with patient and provider-facing components. It was developed and refined following a rigorous, iterative process of user-centered design to support people with HIV receiving outpatient care [15]. Program implementation among a cohort in Central Virginia where the intervention was developed and refined has demonstrated long-term usage and significant improvement in clinical outcomes at 1 [16], 2 [17], and 3 years [18]. The platform has been adapted for other chronic conditions, end users, and contexts [19-24]. To date, *PositiveLinks* has been implemented as part of routine clinical care at 9 clinics in Virginia, and 8 sites in other states, and is considered an evidence-based intervention for HIV care by several national consensus guidelines [25-28]. Clinical effectiveness of PositiveLinks is currently being tested against usual care in a hybrid effectiveness-implementation trial, the *PositiveLinks* in DC Cohort Study, using a cluster randomized controlled trial design (ClinicalTrials.gov NCT04998019) [29]. The trial is being conducted among sites in the DC Cohort Longitudinal HIV Study (DC Cohort Study) following over 12,800 people with HIV at 14 outpatient HIV practice settings, including Federally Qualified Health Centers and academic medical centers [30]. The DC Cohort context for this trial, including cohort site characteristics, as well as study design, site selection, randomization, recruitment, data collection, and statistical analysis procedures, is outlined in the study protocol paper [29].

For complex DHIs like *PositiveLinks* engaging multiple end users collectively within an ‘implementation climate,’ it is important to establish readiness for the coordinated actions needed to implement the intervention across the organization. The theory-based measure, Organizational Readiness for Change (ORIC), was designed and validated by Weiner et al [31] to measure collective readiness for implementation of health care innovations. We opted to measure organizational readiness at each intervention site, including the survey items within provider baseline surveys, in order to understand (1) which relatively lower-scoring sites may require additional support or attention during implementation and (2) to understand at the back end postimplementation if baseline readiness was an influencing factor in provider adoption of *PositiveLinks*. To this end, we also assessed baseline provider usage of mHealth or digital health tools to understand how this experience might shape provider adoption of a new tool within each ‘implementation climate.’

We share our process to proactively define, prioritize, and operationalize evaluation of relevant implementation outcomes and determinants for this type II hybrid effectiveness-implementation trial [4] testing a complex DHI among six DC Cohort sites randomized to the intervention over a 12-month study period. We highlight the unique opportunities and challenges that emerged for planning of a hybrid DHI trial among a large-scale epidemiologic cohort study population. This manuscript shares a practical set of takeaways and considerations that stood out to us as novel or distinct from the available literature we reviewed as we prepared for our DHI trial, that is, what we ‘wish we knew’ before embarking on our extensive planning stage. Several lessons are considerations for teams that would ideally be incorporated as early as the study conception and design stage. Finally, we share the results of an assessment of provider baseline technology usage and pretrial readiness to implement the intervention across participating sites and discuss how results may inform posttrial analyses of implementation outcomes and determinants.

## Methods

### Study Team and Process Refinement

The hybrid trial planning phase spanned an 18-month period preceding onboarding of the first site in December 2022, conducted by an interdisciplinary research team. Research team members hold an established record of clinical research experience, including conducting formative evaluations and observational studies testing clinical efficacy of *PositiveLinks*. Investigators’ primary expertise includes clinical psychology, program evaluation, qualitative methods, instructional design, software development, and implementation research. Program managers contributed empirical observation of *PositiveLinks* implementation processes over a decade that assisted with conceptualization of the intervention, implementation contexts, and components of the implementation strategy. The trial planning team also included DC Cohort Study investigators with expertise in epidemiologic and intervention studies at the cohort sites. The methodological approach to proactively integrate implementation evaluation activities within the hybrid trial required iterative steps conducted through multiple cycles of team feedback, consensus, and refinement (Checklist 1).

### Specify Components of the DHI and Associated Implementation Strategy

Given the multifeature, multiuser nature of the intervention, we created a specified list of components of the DHI implementation process and discrete steps of the implementation strategy. This process was informed by team experience with implementation of *PositiveLinks* in other contexts as part of usual care, including a prior rigorous qualitative study summarizing key in-clinic processes necessary for *PositiveLinks* implementation [32], and a formative preimplementation study engaging stakeholders within the DC Cohort Study context to tailor the app and implementation strategy [33]. Implementation strategy steps were further specified in terms of actors, corresponding actions, and action targets mapped specifically to the DC Cohort Study context [34,35].

### Select Appropriate Implementation Science Frameworks to Accomplish Evaluation Aims

We identified aims for the implementation evaluation arm of the hybrid trial: (1) define and measure implementation outcomes of interest and (2) elucidate determinants of implementation in a rapid, scaled fashion across participating sites. We first used narrative reviews of theories, models, frameworks, and strategy categorization to assess the most widely used determinant and evaluation frameworks [36,37]. We then reviewed technology-specific compendia and original research studies reconceptualizing broader health service frameworks toward DHIs, including hybrid study designs [2,5,6,8,10]. We subsequently selected the RE-AIM evaluation framework and CFIR determinant framework for our first and second implementation evaluation aims, respectively.

### Map Framework Dimensions, Domains, and Constructs to Steps of the Implementation Strategy

We mapped each dimension of the evaluation framework, RE-AIM, to specified components of the intervention and implementation strategy steps, informed by prior efforts in the literature [2,6]. We prioritized measurement of specific steps based on impact of findings for informing future *PositiveLinks* implementations in this context and others, and the feasibility of measurement within the hybrid trial study. For implementation determinants, we selected salient domains/constructs identified from our prior detailed qualitative CFIR-guided assessment of several sites implementing *PositiveLinks* [32]. We also identified salient CFIR interview guide questions for conversion into survey items, which could be rapidly deployed among a larger sample of up to 50 or more cohort providers employed across trial intervention sites at multiple predefined time points (baseline, 6 months, and 12 months).

### Modify or Create Instruments to Support Data Collection for Implementation Outcome Measures and Determinants

Existing data collection instruments developed for the DC Cohort Study or for prior *PositiveLinks* real-world implementation were modified with additional items or created *de novo* within Research Electronic Data Capture to completely assess each RE-AIM implementation outcome measure and salient implementation determinants. Provider survey items were generated using close-ended questions (eg, dichotomous or Likert scale responses), as well as optional free-text responses, and planned for distribution at 6- and 12-months into implementation. Semistructured interviews and focus groups were planned to elicit more detailed feedback post-implementation among a smaller subset of both provider and patient participants, respectively, with guides designed using CFIR.

### Develop a Compatible Data Collection and Management Plan for Implementation Evaluation

Finally, we generated an overall plan for participant data collection and management that ensured compatibility between the clinical effectiveness arm of the trial and the implementation evaluation. Plans for implementation outcome or determinant data collection and abstraction were incorporated into patient approaches for follow-up, study monitoring, and data abstraction already planned for the trial’s effectiveness arm. Provider-related activities specific to the implementation evaluation were incorporated into study onboarding processes.

### Measurement of Baseline Provider Technology Usage and Implementation Readiness

Providers completed an electronic baseline assessment upon enrollment in the study with items related to familiarity, knowledge, and usage of available patient and provider-facing mHealth tools, apps, and portals in routine HIV care, probing specific experience with mHealth tools sharing functionality with components of the *PositiveLinks* intervention. The ORIC 12-item tool [31,38,39] assesses organizational members’ shared resolve to implement a change (change commitment) and shared belief in their collective capability to do so (change efficacy). The ORIC was distributed as part of the provider baseline survey to provider participants at intervention sites.

### Analysis

Descriptive statistics were used to analyze responses to provider survey items related to baseline technology usage and implementation readiness (close-ended response options or free-text responses). Each ORIC item is rated on a 5-point ordinal scale (1=“disagree” to 5=“agree”). Responses were analyzed for frequency, median value, or free-text content as appropriate. ORIC scores were characterized using medians and IQR for total scores (sum of all items) and individual subscores related to change commitment (n=5 items) and change efficacy (n=7) [38]. Statistical analysis was conducted using R version 4.1.2 (R Foundation for Statistical Computing).

### Ethical Considerations

Ethical approval was obtained upon human subject research ethics review by the George Washington University Institutional Review Board (IRB) (Protocol NCR202829; ClinicalTrials.gov NCT04998019) and site-specific IRBs as required. Written study-specific informed consent for all procedures of this trial was collected in addition to the consent obtained for inclusion of clinical data among patient participants upon enrollment in the DC Cohort Study. Written informed consent was obtained for all trial provider participants using procedures outlined in the IRB-approved study protocol. Data collection, storage, and management followed all outlined procedures (eg, deidentification of baseline survey data and use of an assigned study ID with a separate link long). No monetary compensation was provided to participants for study activities described in this paper.

## Results

### Key Process Steps and Takeaways

**Figure 1.**
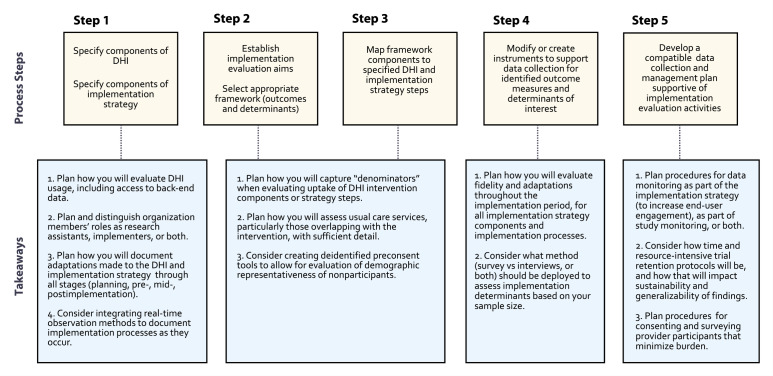
This figure summarizes the 5-step process to plan an implementation evaluation within a hybrid trial for prospective testing of a digital health intervention (DHI). Process steps are outlined, along with respective generalizable takeaways for research teams planning comprehensive implementation evaluations for trials testing DHIs.

Our hybrid DHI trial planning process is summarized in Figure 1, including key process steps and takeaways for research teams aiming to perform similar prospective hybrid trials in real-world settings, where complex DHI implementation and associated study procedures can be planned in advance. We highlight takeaways that emerged from our experience and lacked more applied experience or specific guidance within the literature.

### Define Components of Intervention and Implementation Strategy

Step 1 of the planning phase included specification of *PositiveLinks* intervention components (both patient and provider-facing elements) as well as the respective paradata generated by usage of each feature. The *PositiveLinks* platform contains a large amount of backend data (often described as metadata or paradata) that can be analyzed individually for every participant by feature and is accessible to the study team for analysis. Specific paradata were defined by end user group and data format (individual activity reports over selected time intervals with frequency of use, or free-text post content) in order to plan metrics of uptake for related implementation outcomes defined in steps 2 and 3. Takeaway 1: Research teams should consider during the planning stage what backend data is directly accessible for tools they are studying early in the planning process. Other pragmatic DHI trials may test commercially available tools developed by external companies or vendors, necessitating data-sharing agreements with vendors or suppliers of these tools, proactive design of trial assessments that allow participants to self-report usage at regular (eg, weekly) intervals, or integration of other commercially available tools to track DHI usage into study budgeting, design, consenting, and data collection processes.

**Figure 2.**
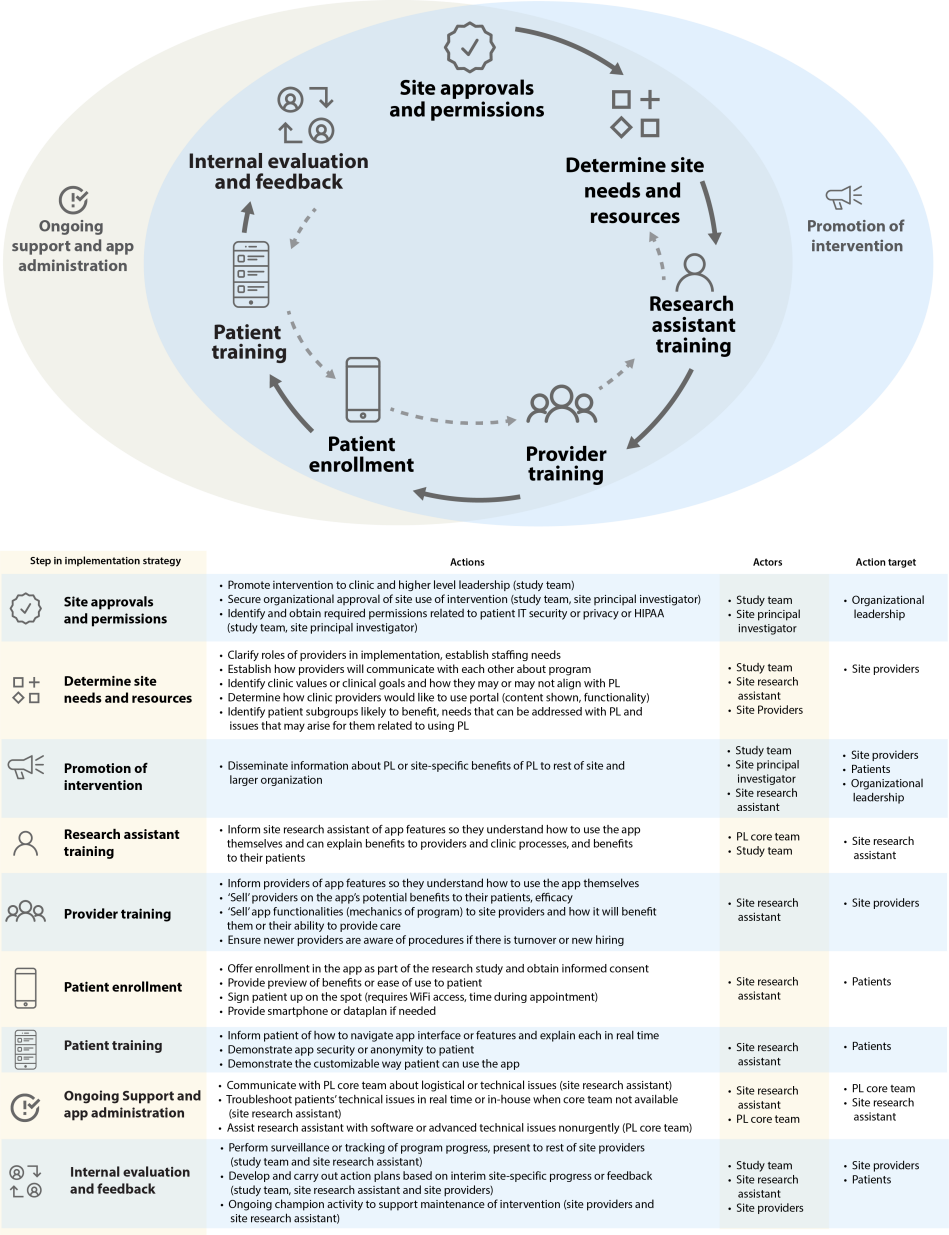
Discrete implementation strategy steps expected for program deployment at DC Cohort sites are demonstrated in the figure and described, specified by step, actions, and actor in the table below it. The arrows show the sequential nature of the activities, but also note the gray dotted arrows indicate the iterative nature of using evaluation and feedback to inform various process steps as implementation proceeds. HIPAA: Health Insurance Portability and Accountability Act; PL: PositiveLinks

Steps of the planned implementation strategy specified for the DC Cohort context are shown in Figure 2. “Actors” include the *PositiveLinks* core team’s program managers who assist remotely with implementation activities and troubleshooting at partner sites. The study team includes researchers involved with coordinating the specific intervention study being conducted to test *PositiveLinks* in the DC Cohort. Study team members were planned to have a larger role in site onboarding processes (determine site needs and resources, conduct research assistant and provider training) within the hybrid trial when compared to real-world implementation. Research assistants employed at DC Cohort Study sites are familiar with recruiting and consenting patients, uploading relevant patient data, and conducting assessments throughout the study period. Site research assistants were thus selected as actors to conduct several steps of the implementation strategy in this context, whereas in real-world implementation, the program is administered by individuals with a range of roles, typically identified by leadership or self-identified as the site lead for implementation. This was a decision made across sites given the extensive role and protected time research assistants have to assist with studies. Takeaway 2: Research teams designing or planning hybrid trials must consider “actors” both in terms of their research roles and implementation roles. If implementation activities in a trial are primarily assigned to a research team member, organization members already responsible for existing usual care activities overlapping with the intervention (eg, for *PositiveLinks*, retention-in-care activities) should also be engaged as early as possible. Shifting more implementation responsibilities to a research team member reduces the pragmatic nature of the trial and challenges intervention sustainability, as teams must decide who will execute those responsibilities when the trial is over.

Distinctions between real-world implementation processes and those expected for the trial emerged at this planning stage. The learning management system (LMS) is a series of interactive online training modules developed for providers and staff, aimed primarily at increasing knowledge and familiarity with the use of platform features (followed by a posttraining feedback survey). Prior to site onboarding for the study, several cohort site principal investigators expressed they would expect significantly lower enrollment of providers if LMS completion was mandatory, primarily due to significant provider clinical burden or competing demands. As a result, participating providers could opt out of LMS completion but were expected to undergo group onboarding or one-on-one training with the site research assistant prior to study participation. Takeaway 3: Research teams should consider incorporating a process to regularly document throughout the pre-, mid-, and postimplementation period, how their specific instance of DHI implementation for the trial diverges from other implementations, adaptations, or real-world practice, representing fidelity of implementation through every stage (not just during the intervention implementation period).

Real-world *PositiveLinks* implementation offers collaboration between sites in a “Community of Practice,” to share implementation challenges, solutions, and unique adaptations to improve patient experiences and engagement. In contrast, we sought to maintain internal validity and avoid cross-site contamination for the trial, so intervention sites were not included in the “Community of Practice.” Additionally, steps like administration of as-needed technical app support/troubleshooting and promotion of the intervention are usually more informally evaluated through site discussions and internal meetings during real-world implementation. During planning, we determined they would be challenging to evaluate with structured tools and opted to evaluate them through qualitative interviews at completion of the study. Takeaway 4: Clinic-embedded DHIs implemented in real-world practice can involve complex interactions between actors both across sites and within sites, and over time. Reserving evaluation of these processes to the late stages of implementation can result in missed opportunities to capture important real-world factors influencing implementation. Within the constraints of budget and time, research teams should consider inclusion of study methods to obtain real-time observations of these implementation processes as they occur. These could include direct observations (with field logs or notes) by implementers (providers, RAs), and small “check-ins” or “debriefs” conducted by a study team member throughout the study period.

### Select Appropriate Frameworks and Map Components to Implementation Strategy Steps

Output for steps 2 and 3 are summarized in Table 1. Specifically, RE-AIM evaluation framework dimensions were mapped to each prioritized step within the implementation strategy. During this step, we identified discrete datapoints that required collection to assess each RE-AIM dimension. Other DHI hybrid trials with detailed reporting of framework app describe similar approaches, aligning DHI usage and other aspects of program uptake by patients and providers with reach and adoption dimensions, respectively [6,32]. Several unique takeaways emerged in our effort.

**Table 1.** RE-AIM^a^ framework mapped to intervention/implementation strategy, corresponding outcome measures, data collection methods^b^.

RE-AIM dimension	Outcome measures	Data source or data collection instrument
Reach: *How do I reach the targeted population with the intervention?*	Number of patients offered enrollment/number seen at site	DC Cohort Database, DC Cohort Study Patient Consent Logs
	Number of patients completing enrollment/number offered	PL^c^ Platform Paradata, DC Cohort Study Patient Consent Logs
	Number of patients accessing PL app/number enrolled	PL Platform Paradata
	Sociodemographics of patients enrolled (representativeness)	DC Cohort Database
	Sociodemographics of patients declining enrollment (representativeness)	DC Cohort Study Patient Consent Logs
Effectiveness: *How do I know my intervention is effective?*	HIV viral load suppression (<200 copies/mL)	DC Cohort Database
	Visit constancy	DC Cohort Database
	Retention in care	DC Cohort Database
	Demographics for patients meeting efficacy end points versus those not meeting	DC Cohort Database
	PL usage patterns for patients meeting efficacy end points versus those not meeting	PL Platform Paradata
Adoption: *How do I develop organizational support to deliver my intervention?*	Number of providers who completed onboarding/number employed	Provider Baseline Survey, DC Cohort Site Assessment Survey (Multimedia Appendix 1)
	Number of providers who completed LMS^d^/number enrolled	PL Posttraining feedback survey
	Number of providers accessing PL app/number enrolled	PL Platform Paradata
	Demographics for providers	Provider Baseline Survey (Multimedia Appendix 2)
	Cohort site characteristics	DC Cohort Site Assessment Survey
Implementation: *How do I ensure the intervention is delivered properly?*	Dose, fidelity, and adaptations: patient training	Provider Follow-up Survey (Multimedia Appendix 3), Patient Interview Guides
	Dose, fidelity, and adaptations: provider training	PL Posttraining feedback survey, Provider Interview Guides
	Dose, fidelity, and adaptations: provider PL usage	PL Platform Paradata, Provider Follow-up Survey, Provider Interview Guides
Maintenance: *How do I incorporate the intervention so that it is delivered over the long term?*	Patient intent/interest to continue using PL following trial completion	Patient Interview Guides
	Provider intent/interest to continue using PL following trial completion	Provider Follow-Up Survey, Provider Interview Guides

a RE-AIM: Reach Effectiveness Adoption Implementation Maintenance.

b Data collection methods include abstraction from one or more items from existing data sources, modification or addition of items to existing data collection instruments, or creation of new instruments. Visit constancy is the proportion of 4-month intervals a visit is completed in 12 months. Retention in care per HRSA-1 definition: 2 appointments attended at least 90 days apart within 12 months.

c PL: PositiveLinks.

d LMS: learning management system.

As we reviewed the reach and adoption dimensions, we conceptualized how we could measure not only absolute numbers as measures of uptake (eg, number of patients/providers who download and use the app), but also estimate proportions: how many people could have taken up the DHI when it was offered at each site? The DC Cohort Site Assessment Survey is periodically deployed to cohort sites for updated assessment of available service delivery. We added several items to this Site Assessment Survey to capture the denominator of people employed for each type of provider role (eg, attending physician, clinic nurse, case manager, social worker, etc) as well as site-level baseline mHealth or technology use (Multimedia Appendix 1) to more adequately assess the adoption dimension for *PositiveLinks* implementation. Takeaway 1: Research teams should consider in advance how they will capture ‘denominators’ for both patients (reach) and providers/staff serving as implementers (adoption), including a need for pretrial site-level data collection that can provide denominators, and with what frequency they need to be deployed surrounding implementation.

Several existing Site Assessment items were identified for abstraction for evaluation of the adoption dimension of RE-AIM (eg, on-site clinical services and support services, updated staffing of clinical and nonclinical providers, specialty training). Takeaway 2: Research teams testing DHIs across multiple care settings must consider how they will sufficiently characterize existing “usual care” services available, which can vary widely across care settings and change over time. This step is particularly important for DHIs that do not study direct-to-consumer tools, but instead require DHIs to become integrated into, or penetrate, an implementation climate and existing practices. Understanding to what extent the tool is serving as an adjunct or supplementary tool layered on top of an already robust organizational usual practice, versus creating a service where none existed, is crucial for interpreting and contextualizing effectiveness findings from a hybrid trial, in addition to other implementation outcomes.

A distinct opportunity also arose from conducting the hybrid trial among the DC Cohort Study. DC Cohort patients who choose not to participate in the *PositiveLinks* program at intervention sites have already consented to inclusion of their data in the cohort database, so these nonparticipants could be examined to understand sociodemographic representativeness, a component of patient “reach” within RE-AIM that appears infrequently in published applications of this framework. Patient consent logs standardized for the DC Cohort Study include patients’ reasons for declining participation, with up to 3 approaches. These logs were modified to query a selected number of demographics for cohort participants declining to participate in the study (age, sex, race, ethnicity, insurance status, last CD4 count, and last HIV viral load). Takeaway 3: Teams designing DHI trials outside of existing cohorts with available data should consider use of preconsent tools that collect deidentified demographics of interest among clinic patients who decline the intervention, in order to evaluate this understudied component of patient reach.

### Modify or Create Instruments to Support Data Collection for Implementation Outcome Measures and Determinants

Our process yielded several strategies to support data collection of all identified implementation outcomes: (1) *creation* of new instruments for prospective data collection specific to the implementation evaluation, (2) *modification* of standardized tools used for DC Cohort intervention studies or *PositiveLinks* evaluations or (3) *abstraction* from existing *PositiveLinks* or DC Cohort sources (eg, DC Cohort Database storing patient encounter, laboratory, and sociodemographic data). Modification and abstraction planned based on steps 2 and 3 from existing data sources are described previously.

Instruments created specifically for the implementation arm of the hybrid trial included surveys directed at providers not usually targeted by data collection for intervention studies at cohort sites. The provider baseline survey (Multimedia Appendix 2) and follow-up survey (Multimedia Appendix 3), annotated with respective framework components, were designed to capture otherwise unincorporated data points for measurement for the implementation dimension of RE-AIM (eg, fidelity, dose, and any adaptations made to steps of the implementation strategy by site providers or research assistants). Many DHIs require a complex set of steps to ensure both patient and provider engagement. For *PositiveLinks,* this included providers themselves understanding the app features, how they work and their impact, then remembering and being motivated to bring up the app and promote it during a routine clinical encounter, be able to describe its features and benefits, and refer the patient to a staff member (eg, research assistant). The research assistant must then effectively assist the patient to download the app, register for the account, and train them on its usage (Figure 2). The provider follow-up surveys were thus designed to capture the extent to which providers performed each of these tasks over multiple time points (Multimedia Appendix 3), in addition to questions probing self-rated fidelity and adaptations made to the use of the tool and its features over multiple time points. Takeaway 1: Comprehensive evaluations of DHIs should consider fidelity and adaptations of aspects of implementation beyond direct end user engagement with the digital tool, and data collection instruments may consequently need to be created to assess implementation outcomes related to these specific steps over multiple time points.

Salient implementation determinants adapted for inclusion from our prior CFIR-guided rapid evaluation study of *PositiveLinks* real-world implementation were: inner setting (compatibility), outer setting (patient needs and resources, external policy, and incentives), characteristics of individuals (knowledge and beliefs), innovation characteristics (adaptability and complexity), and implementation process (planning and engagement of key stakeholders). Postimplementation patient focus group and provider in-depth interview guides were also adapted from prior *PositiveLinks* implementations using salient CFIR 1.0 domains or constructs. Takeaway 2: Surveys designed using CFIR or other determinant frameworks offer an opportunity to more rapidly probe a wide array of domains and constructs among a larger sample, but should be planned in conjunction with richer data collection methods (qualitative). The surveys we designed were limited to previously identified salient constructs during our rapid evaluation study in other contexts (cite), and if used alone would miss important contextual factors for this trial.

### Develop a Compatible Data Collection and Management Plan for Implementation Evaluation

Finally, we developed a plan to specify timing and frequency of data abstraction (eg, from the DC Cohort Database) and collection in relation to planned activities for the effectiveness arm of the trial (eg, patient consent or enrollment, administration of baseline, 6 mo, and 12 mo assessments). For patient data, plans were designed to minimize separate approaches as well as ‘data pulls’ from existing sources anticipated to support evaluation of clinical effectiveness outcomes. Further, monitoring of feature usage from platform paradata for patients and providers is a routine part of real-world *PositiveLinks* implementation, to guide efforts to engage and re-engage staff and enrolled patients, troubleshoot concerns in real-time, and ensure sustainability. Frequency of paradata abstraction for monitoring was thus predetermined for specific features at timepoints throughout implementation. Takeaway 1: Research teams should consider data monitoring both as a study activity and as a part of the implementation strategy. If regular data monitoring is expected to increase engagement or generate actionable data for implementers, this activity itself becomes a component of the implementation strategy and should both be integrated into program planning and measured for fidelity to plans along with other strategy components. Takeaway 2: Similarly, trial retention protocols whereby participants showing low engagement are contacted by study team members through a series of time and resource-intensive activities ultimately impact intervention uptake (reach and adoption). The design of these protocols should be considered in the interpretation of generalizability/sustainability of observed implementation outcomes.

We required informed consent for all provider activities in the evaluation, including collection of provider survey responses and participation in postimplementation in-depth interviews. Selection of the timing and frequency of provider survey administration required consideration of provider turnover, particularly in participating intervention sites with higher expected turnover (eg, trainees like infectious disease fellows rotating within academic centers). Takeaway 3: Implementation evaluations engage providers as participants, which is distinct from most intervention efficacy trials, and onboarding processes should consider integrating consent procedures and baseline survey administration to reduce the burden and frequency of study procedures on participating providers.

### Baseline mHealth/technology Use and PositiveLinks Implementation Readiness

A total of 17 providers and RAs have completed provider baseline surveys to date. Self-reported mHealth/technology use for various aspects of patient care at baseline is summarized in Table 2. Among the 17 respondents, 9 reported access to a patient messaging feature via their electronic medical record system (EMR). Usage of additional non-EMR messaging tools was reported by 6 providers. Overall satisfaction with non-EMR methods of messaging was high for those reporting usage (n=6), and reported frequency of use was higher for non-EMR tools compared to EMR-based messaging. Fewer respondents completed optional survey items related to non-EMR tools for sharing lab results or exchanging documents with patients, with variable frequency and satisfaction with described tools.

We incorporated the ORIC tool into the provider baseline survey to assess collective baseline readiness at each site [31]. The median total ORIC score for all providers was 48 (IQR 45‐54, possible score range 12‐60), with RAs scoring slightly higher than physicians (52.5 (44-55) vs 48.0 (46-49)). Median total scores for change commitment and change efficacy were 20 (IQR: 19‐22, possible score range 5‐25) and 28 (27‐30, possible score range 7‐35). Total change efficacy scores were slightly higher for RAs compared to providers (30.5 vs 28.0).

**Table 2.** Provider and research assistant baseline usage of mobile health (mHealth) tools by functionality.

Baseline survey questions	Response rate, n (%)
Messaging	
Does your EMR^a^ system have a patient portal that allows you to directly message with your patients? (n=17)	
Yes	9 (53)
No	6 (35)
Not sure	2 (12)
If yes, how frequently do you use it to message with your patients? (n=9)	
Never	2 (22)
Rarely	2 (22)
Occasionally	3 (33)
Frequently	2 (22)
Very frequently	0 (0)
Please specify name of apps or tools or websites used to message patients (n=6)	
Ring central	2 (33)
Halo	1 (17)
EMR mobile or web-based platform	1(17)
Text messaging	2 (33)
In the past 3 months, how many times have you used this app/tool/site to message patients? (n=6)	
Never	0 (0)
Rarely	0 (0)
Occasionally	1 (17)
Frequently	4 (67)
Very frequently	1 (17)
To what extent are you satisfied with these telemedicine services used for messaging? (n=6)	
Very unsatisfied	0 (0)
Unsatisfied	0 (0)
Neutral	0 (0)
Satisfied	4 (67)
Very satisfied	2 (33)
Laboratory results	
Please specify name(s) of app/tool/website(s) to share lab results. (n=3)	
EMR autosend letter	1 (33)
EMR mobile or web-based platform	1 (33)
Clinic phone	1 (33)
In the past 3 months, how many times have you used this app/tool/site to share laboratory results? (n=3)	
Never	0 (0)
Rarely	0 (0)
Occasionally	1 (33)
Frequently	1 (33)
Very frequently	1 (33)
To what extent are you satisfied with this app or tool or website for sharing laboratory results? (n=3)	
Very unsatisfied	0 (0)
Unsatisfied	0 (0)
Neutral	0 (0)
Satisfied	2 (67)
Very satisfied	1 (33)
Documents	
Please specify the name of app or tool or website to share or receive documents (n=2)	
Encrypted email	1 (50)
EMR mobile/web-based platform	1 (50)
In the past 3 months, how many times have you used this app/tool/site to share or receive documents? (n=2)	
Never	0 (0)
Rarely	0 (0)
Occasionally	1 (50)
Frequently	1 (50)
Very frequently	0 (0)
To what extent are you satisfied with this app or tool or website for sharing or receiving documents? (n=2)	
Very unsatisfied	0 (0)
Unsatisfied	1 (50)
Neutral	0 (0)
Satisfied	1 (50)
Very satisfied	0 (0)

a EMR: electronic medical record.

**Figure 3.**
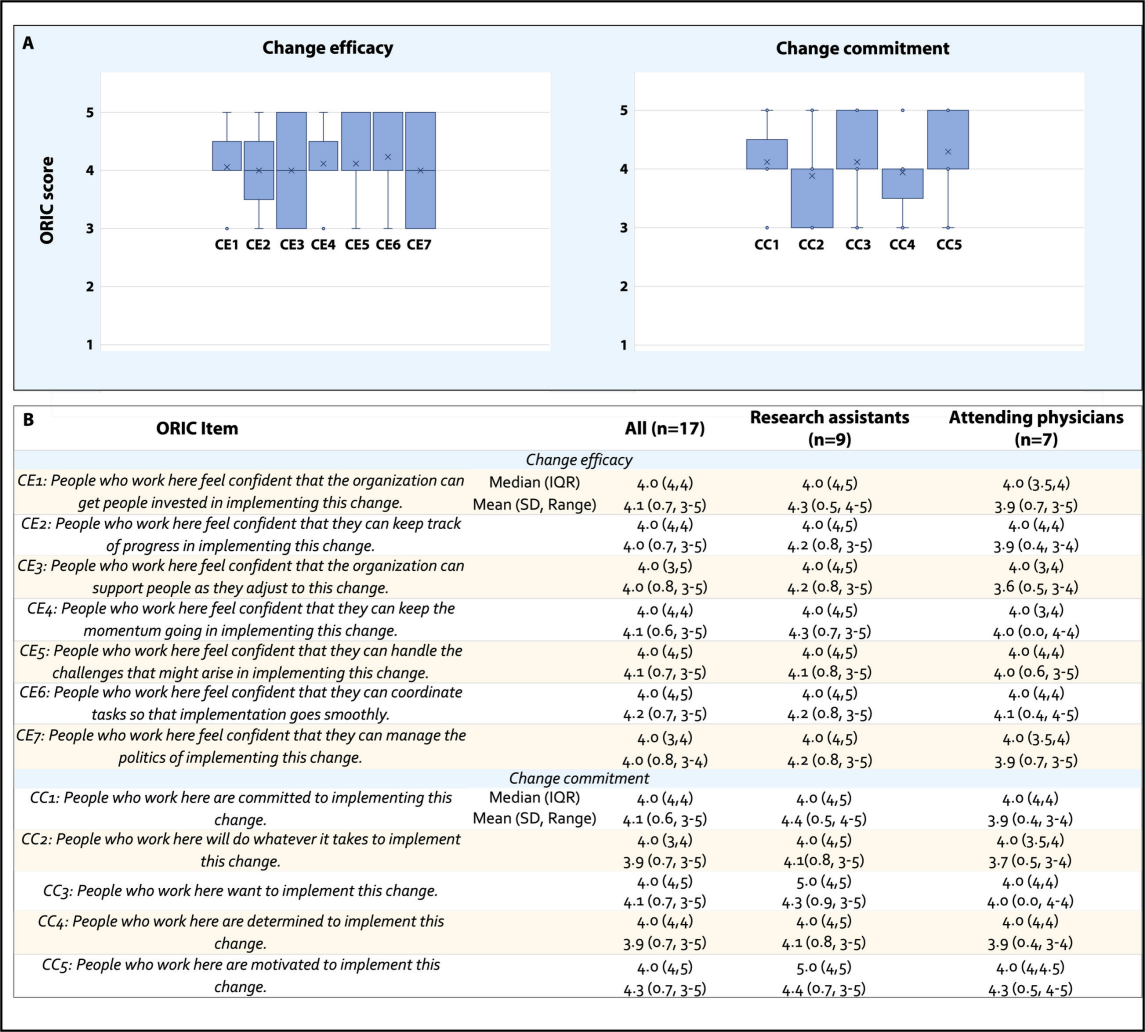
Baseline ORIC scores summarized for all respondents (n=17) at 6 sites randomized to implement the intervention (A) as well as by respondent type such as research assistants and attending physicians (B). One social worker completed the survey but ultimately withdrew from the program. ORIC: Organizational Readiness to Implement Change.

Individual items corresponding to change efficacy (CE1-CE7) were scored on average between 4 and 4.2, with median scores of 4 (IQR 3-4) for all items (“Somewhat agree”), and no items scoring lower than 3 (“Neither agree nor disagree”; Figure 3). All change efficacy scores ranged from 3 to 5 except for CE7 (‘People who work here feel confident that they can manage the politics of implementing this change’), which along with CE3 (”People who work here feel confident that the organization can support people as they adjust to this change”) had a distribution with greater spread due to relatively higher frequency of neutral or “3” scores.

ORIC items corresponding to change commitment (CC1-CC5) similarly scored with a median of 4 (IQR 3-5) for all items (Figure 4), means ranging from 3.88 to 4.2, and scores also ranged from 3 to 5 for all items. Change commitment score distributions were slightly more negatively skewed for items CC2 (“People who work here will do whatever it takes to implement this change”) and CC4 (“People who work here are determined to implement this change”). When analyzed by provider type, mean scores were higher across all individual ORIC items for RAs compared to physicians. Median scores within subgroups were 4 (with IQR values ranging from 3.5 to 5, Figure 4) for a majority of items except for two change commitment items with higher median scores for RAs: CC3 (“People who work here want to implement this change”) and CC5 (“People who work here are motivated to implement this change”).

## Discussion

### Principal Results

We describe a study team’s experience planning and proactively integrating implementation science methodology into a hybrid DHI study conducted within an existing cohort study. This experience included a process to proactively define, prioritize, and operationalize measurement of implementation outcomes salient to a prospective DHI hybrid trial, as well as development of data collection instruments compatible with rapid assessment of implementation determinants and outcomes at scale for a large cohort and over multiple time points throughout the trial. Our experience highlights the limitations of prospective “pragmatic” trial designs to reflect real-world DHI implementation, and the unique opportunities and challenges of DHI trial planning within a large-scale cohort study population. We assessed baseline provider mHealth or technology usage and organizational readiness at baseline, which will be applied as a site-level characteristic or covariate during exploratory analysis of differential site and provider-level adoption of the intervention, including specific features, throughout the implementation period. We predict findings may align with site-specific implementation determinants revealed by CFIR application (eg, innovation relative advantage) during qualitative analysis, as well as account for variation in implementation outcome measures (eg, adoption by platform feature) across sites where providers and research assistants, for example, were already frequently using existing digital tools overlapping with *PositiveLinks* functionality that were fully integrated into their clinic’s EMR with a high degree of satisfaction and had high ORIC scores. Readiness assessment results varied across sites but were not sufficiently different to prompt differential approaches to implementation support across sites. Results suggest that among the providers and RAs queried at the 6 intervention sites preimplementation, belief in their sites’ collective capability to implement the program was high overall, particularly in relation to internal abilities to coordinate tasks and handle challenges arising within the sites. However, respondents’ confidence was lower in external actors’ influence on implementation, or the organizational politics and support necessary to execute program components. To increase collective change efficacy of providers and research assistants, concerted preimplementation evaluation efforts may be needed in future *PositiveLinks* implementations to build site members’ confidence that higher organizational levels of support for the program are present.

### Comparison With Prior Work

To apply relevant implementation science frameworks to DHI implementation evaluation, we reviewed how investigators compared Proctor’s Outcomes for Implementation Research recategorized for DHIs against RE-AIM [2,6]. These and other original research studies had limitations including a lack of measurable eligible “denominators” available for certain outcomes (eg, provider adoption dimension of RE-AIM). Prior studies also focused on conceptualizing outcomes for a primarily patient-facing intervention itself (eg, different ways of leveraging back-end usage capture data and tracking referrals of patients by clinic to the study), rather than including additional strategy steps to implement a dual-facing (patient and provider) intervention (mapped in Figure 2), which require dedicated measurement of both patient and provider inputs.

While RE-AIM offered adequate operational guidance to evaluate this DHI and implementation strategy as with some prior studies [2,6], several takeaways, opportunities, and challenges arose throughout our planning process as detailed throughout this article and summarized in Figure 1. Since the completion of our planning process and preparation of this manuscript, a framework for designing DHI hybrid trials has been published [32], which shares similarities with our identified planning process steps (specify components of the digital intervention as well as support services and implementation strategy, delineate domains being studied, ie, actors and action targets). Our takeaways delve deeper into considerations researchers should make based on specific experience with: (1) designing a randomized trial where the digital intervention is tested against a usual care condition, contains multiple features and engages both patients and providers or organization members; (2) integrating understudied components of RE-AIM and related evaluation frameworks into DHI trial design (eg, representativeness, fidelity, dose, and adaptation); and (3) conducting a trial among an epidemiologic cohort.

There is a well-recognized tension between maintaining the rigor and validity of randomized trial designs and establishing pragmatic conditions more relevant to real-world implementation [40]. By performing a preimplementation planning phase for a prospective DHI trial that incorporated frameworks proactively, we ensured collection of a robust set of quantitative and qualitative data. In contrast, post hoc evaluations conducted in other studies available in the literature are frequently reported among “real world” conditions or ‘usual care’ expected of implementation research as most strictly defined. When not planned in advance for inclusion, there are significant limits on the extent to which additional implementation outcomes like fidelity, dose, program adaptations, and uptake of specific components or steps of implementation have been evaluated with sufficient granularity and coverage of participants over the course of implementation [41,42] in these studies. Simultaneously, however, tighter control of prospective hybrid trial conditions and administration of the program through protocolized research activities inherently limits how well ‘real-world’ or pragmatic conditions are reflected within a hybrid trial.

### Limitations

Several limitations emerged within our process. This more comprehensive planning process for the implementation evaluation was undertaken following receipt of funding for the award, which is common for hybrid trials. We found that ideally, this process should be undertaken as early as possible. Several takeaways from our planning process require attention this early, impacting study design and planned procedures, and consequently even impacting study budgeting and scope of work for research staff.

We noted several key limitations regarding the intended pragmatic nature of our prospective hybrid DHI trial. Real-world *PositiveLinks* implementation often relies on outreach by partner sites, by individuals who serve as champions of the intervention with an active role in obtaining site approvals (related to data security and patient privacy), and who continue to promote the intervention. Research assistants were assigned as de facto program managers at sites participating in the hybrid trial; however, this is a major distinction from real-world implementation. This decision, while needed to rapidly plan and conduct a multisite trial within budgetary and time-related constraints, represents a tradeoff in terms of generalizability of this planned prospective *PositiveLinks* implementation research when the intervention requires a distinct, multilayered ‘implementation climate’ and ‘champions’ within that climate. These ‘climates’ typically require gradual building of multiple, interacting implementers’ self-efficacy, motivation, and longitudinal intervention promotion efforts to ensure site readiness and penetration.

Additional considerations for hybrid trials implementing DHIs may represent challenges to generalizability in terms of real-world maintenance and sustainability, including providing technology to patients (eg, smartphones, data plans), incentives for usage of the intervention or specific features, and participant retention protocols common for clinical efficacy trials. Real-world PositiveLinks implementation variably includes provision of phones and data plans, depending on specific partner site funding availability and patient need, and re-engagement protocols are also a routine part of implementation at several sites. For this trial, we planned to rely on existing site-specific or federal subsidy programs available to participants in the context (eg, Federal Communications Commission Lifeline program) before providing smartphones and data plans, and a retention protocol was used by site RAs to periodically re-engage patients. No additional incentives were planned for higher levels of app usage, however, and patients who do receive smartphones can keep them for the duration of the study regardless of app usage.

Examining implementation in parallel with a cluster randomized effectiveness trial among an epidemiological cohort presented another set of unique challenges and opportunities. This hybrid approach allows for a scaled implementation evaluation to occur across multiple sites simultaneously, leveraging existing research infrastructure, including site staffing with research assistants and existing data collection instruments. The scaled, simultaneous multisite approach, however, engages a larger number of patients and providers within time and funding constraints of a single hybrid trial and necessitates consideration of more rapid, cost-effective approaches to implementation evaluation (acknowledged by de la Vega et al [6]). Provider surveys, for example, were designed to capture implementation outcome measures and determinants for the larger expected sample of respondents at 6 intervention sites than are typically engaged with more in-depth qualitative processes applying these frameworks, in particular CFIR. There is no consensus in the implementation science field about how these frameworks reflecting complex psychosocial/behavioral constructs should be applied, including whether to attempt to dichotomize or categorize items for broader, rapid distribution. Surveys could, for example, introduce study team bias during creation and selection of specific items to probe and limit more systematic application of the framework [37]. Combining surveys and qualitative approaches can offer opportunities to validate the latter methods, but more extensive psychometric validation of survey tools is needed to ensure generalizability and validity. Finally, readiness assessment findings are based on self-reported survey items, subject to scoring biased by the individuals’ level of involvement with pretrial planning and other procedures (eg, research assistants vs physicians).

### Conclusions

Implementation research for complex DHIs can expand understanding of how these interventions will behave in “real world” conditions. Prospective hybrid effectiveness-implementation trials can facilitate more in-depth implementation evaluations at scale if appropriately planned. Our experience highlights the ways in which evaluations must attempt to balance rigor, proximity to “real world” implementation climates, and incorporate multiple key implementation outcomes and determinants within the time and resource constraints of a prospective DHI hybrid trial. Based on our experience, planning processes for hybrid DHI trials should include:

- Specification of discrete DHI and associated implementation strategy components, considering how end users will engage with each, and what study procedures should be planned and budgeted to adequately measure that engagement (eg, backend paradata).
- Strategies to observe and document adaptations and real-time implementation processes throughout the planning, pre-, mid-, and postimplementation periods.
- Plans ahead of time to capture denominators of uptake outcomes (reach for patients and adoption for providers), demographic representativeness within those reached by the DHI versus not, and a method to capture usual care services that overlap with DHI functionalities across trial sites.
- Plans to evaluate fidelity and adaptations to the DHI and implementation strategy steps carried out among the implementing organization site that go beyond the use of the tool itself.
- Considerations for how to obtain detailed descriptive data related to implementation determinants in a larger sample size of participants (eg, design survey tools, interviews, or both using determinant frameworks).
- Plans for study procedures that minimize provider or implementer burden but enable consenting, data monitoring, and surveying of those providers, and consider in protocol design how retention protocols and use of research staff over “usual care” staff challenges generalizability or sustainability.

## Supplementary material

10.2196/76327 Multimedia Appendix 1 DC cohort site assessment survey with modifications.

10.2196/76327 Multimedia Appendix 2 Provider baseline survey.

10.2196/76327 Multimedia Appendix 3 Provider follow-up survey.

10.2196/76327 Checklist 1 CONSORT-EHEALTH (V 1.6.1) checklist.

## Abbreviations

CFIR: Consolidated Framework for Implementation Research
DHI: digital health intervention
EMR: electronic medical record
IRB: institutional review board
LMS: learning management system
mHealth: mobile health
ORIC: Organizational Readiness to Implement Change
RE-AIM: Reach Effectiveness Adoption Implementation Maintenance
