# Supplementary material for: A Complex Digital Health Intervention to Support People With HIV: Organizational Readiness Survey Study and Preimplementation Planning for a Hybrid Effectiveness-Implementation Study
Source: J Med Internet Res. 2026 Jan 21;28:e76327. doi: 10.2196/76327 (PMC12823349; doi:10.2196/76327)
Supplement: Multimedia Appendix 1 [file jmir-v28-e76327-s001.docx]

**Multimedia Appendix 1: DC Cohort Site Assessment Survey with Modifications**

**Existing DC Cohort Site Assessment Survey Items for Implementation Evaluation**

**What types of providers provide HIV care at your DC Cohort clinic? Select all that apply.**

▢ Attending physicians

▢ Fellows

▢ PAs

▢ NPs

▢ RNs

▢ Social workers

▢ Other medical providers

**How many attending physicians do you have overall?** (Text field)

**What proportion of providers are HIV Specialists?** **(e.g. AAHIVM, IAPAC, or infectious disease trained)**

▢ 0% ▢ 1-25% ▢ 26-50% ▢ 51 - 75% ▢ 76-100%

**How many fellows do you train annually?** (Text field)

**What proportion are HIV Specialists? (e.g. AAHIVM, IAPAC, or infectious disease trained)**

▢ 0% ▢ 1-25% ▢ 26-50% ▢ 51 - 75% ▢ 76-100%

**How many PAs do you have overall?** (Text field)

**What proportion are HIV Specialists?** **(e.g. AAHIVM, IAPAC, or infectious disease trained)**

▢ 0% ▢ 1-25% ▢ 26-50% ▢ 51 - 75% ▢ 76-100%

**How many NPs do you have overall?** (Text field)

**What proportion are HIV Specialists**? (e.g. AAHIVM, IAPAC, or infectious disease trained)

▢ 0% ▢ 1-25% ▢ 26-50% ▢ 51 - 75% ▢ 76-100%

**How many RNs do you have overall?** (Text field)

**What proportion are HIV Specialists?** (e.g. AAHIVM, IAPAC, or infectious disease trained)

▢ 0% ▢ 1-25% ▢ 26-50% ▢ 51 - 75% ▢ 76-100%

**How many SWs do you have overall?** (Text field)

**What proportion are HIV Specialists?** (e.g. AAHIVM, IAPAC, or infectious disease trained)

▢ 0% ▢ 1-25% ▢ 26-50% ▢ 51 - 75% ▢ 76-100%

**Please specify types of other medical providers:** (Text field)

**How many other medical providers do you have overall?** (Text field)

**What proportion are HIV Specialists?** (e.g. AAHIVM, IAPAC, or infectious disease trained)

▢ 0% ▢ 1-25% ▢ 26-50% ▢ 51 - 75% ▢ 76-100%

**Select medical specialties available at your DC Cohort site:**

▢ Cardiology

▢ Gynecology

▢ Ophthalmology

▢ Psychiatry

▢ Dentistry

▢ Hepatology

▢ Neurology

▢ Dermatology

▢ Infectious disease

▢ Pediatrics

▢ Gastrointestinal

▢ Oncology

▢ Primary care

▢ Other (Please select all that apply to your site)

**Please describe other medical specialties available at your DC Cohort site**: (Text field)

**Please indicate below whether your DC Cohort clinic offers these services for HIV-infected patients. (Please select all that apply)**

▢ On-site clinical pharmacy

{Branching logic}

For the indicated on site clinical pharmacy, please answer the following four questions below.

Is a patient’s records in the pharmacy database linked to the patient’s records in the patient database at the clinic using a common patient ID?

▢ Yes ▢ No

Is there an electronic database at the pharmacy that tracks the distribution of ART at the patient level?

▢ Yes ▢ No

Does your on site pharmacy offer door to door delivery of medications?

▢ Yes ▢ No

Does your on site pharmacy offer mail order delivery of medications?

▢ Yes ▢ No

▢ Urgent care

▢ Substance abuse counseling

▢ Opioid treatment programs

▢ Case management

▢ Peer intervention programs

▢ Nurse navigation

▢ Job training referrals

▢ Housing referrals

▢ Transportation services

▢ EIA/WB

▢ 3rd generation HIV antibody testing (rapid testing)

▢ 4th generation HIV testing

▢ Other (describe)

{Branching logic}

Please describe other: (Text field)

**Has your clinic ever participated in any of the following DC Dept of Health activities? (Please select all that apply)**

▢ Ryan White Care program

▢ Recapture Blitz

▢ TLC Plus-non incentive site

▢ TLC Plus incentive site

▢ Routine testing

▢ Red Carpet Entry

▢ Positive Pathways

▢ PrEP provider

▢ Needle exchange program

▢ Other (describe)

{Branching logic}

Please describe other services: (Text field)

**Which ART adherence support activities are provided at your DC Cohort HIV clinic? (Please select all applicable responses)**

▢ One-on-one counseling

▢ Group counseling

▢ Written education material like calendars and reminders

▢ Pill boxes or blister packs

▢ Alarm clocks, wrist watches, beepers

▢ Telephone calls/text messages

▢ Routine review of medication pick up

▢ Other (specify)

{Branching logic}

Please describe other: (Text field)

**What practices does your site use to link and retain HIV-infected patients in care? Please select all that apply to your clinic.**

▢ Routine follow-ups on patients who miss their appointments via mail, phone, or other

▢ Providers offer care to persons with any income level and insurance status

▢ Case management

▢ Patient navigation services (e.g., accompany to appointments as needed)

▢ Systematic monitoring of retention in care (e.g. monitoring visit adherence, gaps in care, or visits per interval of time)

▢ Practice uses e-prescribing for auto refills, even if the patient missed visits

▢ Conduct case reviews at regularly scheduled clinical meetings

▢ Procedures in place to coordinate care between HIV clinic staff and providers in other specialty areas (e.g. cardiology, endocrinology)

▢ Other

{Branching logic}

Please describe other: (Text field)

**Added Form Items for Implementation Evaluation**

How many case managers do you have at your clinic overall now? (Text field)

How many peer navigators do you have at your clinic overall now? (Text field)

How many community health workers do you have at your clinic overall now? (Text field)

How many eligibility specialists do you have at your clinic overall now? (Text field)

How many pharmacists do you have at your clinic overall now? (Text field)

**What telehealth platforms was your clinic using prior to the pandemic? (select all that apply)**

▢ None

▢ Doximity

▢ Whatsapp

▢ Clinic EHR

▢ Zoom

▢ Telephone

▢ Other

If other please specify (Text field)

**Does your DC Cohort clinic site use any other mobile Health apps, websites, or electronic tools separate from the electronic medical record system to support patient care? (e.g. tools to assist with care coordination, sharing lab results or documents related to care, arrange opportunities for peer support, provider communication with patients or other providers)**

▢ Yes

{Branching logic}

Please specify the name of the tool(s) (e.g. Facetime, Whatsapp, doodle polls, etc): (Text field)

**Please specify the functionality of the tool(s) (e.g., tools to share labs with patients electronically, calendar tools to share appointment info across staff, doodle polls to coordinate patient group meetings, etc.) (**Text field)

Please specify the functionality of the tool(s) (e.g.tools to share labs with patients electronically, calendar tools to share appointment info across staff, doodle polls to coordinate patient group meetings, etc) (Text field)

Please describe how you access this tool(s) (e.g. website, smartphone app): (Text field)

▢ No

▢ Don’t know
