# Supplementary material for: A Complex Digital Health Intervention to Support People With HIV: Organizational Readiness Survey Study and Preimplementation Planning for a Hybrid Effectiveness-Implementation Study
Source: J Med Internet Res. 2026 Jan 21;28:e76327. doi: 10.2196/76327 (PMC12823349; doi:10.2196/76327)
Supplement: Multimedia Appendix 2 [file jmir-v28-e76327-s002.docx]

**Multimedia Appendix 2: Provider Baseline Survey**

1. **Name** (Your name will be used exclusively for the purpose of tracking characteristics of platform users, and will be un-linked from your responses. Your responses will not be reviewed until de-identified): (Text field)
2. **Age**: (Text field)
3. **What is your gender?**

▢ Female

▢ Male

▢ Transgender male

▢ Transgender female

▢ Nonbinary

▢ Prefer not to answer

1. **Do you consider yourself to be Hispanic or Latino/a?**

▢ Yes

▢ No

1. **Which racial group do you consider yourself? You may choose more than one option.**

▢ Asian

▢ American Indian or Alaska Native

▢ Black or African American

▢ Native Hawaiian or Other Pacific Islander

▢ White

▢ Other

{Branching logic}

If other, please specify: (Text field)

▢ Prefer not to answer

1. **Clinic name:** (Dropdown selection)
2. **What is your role in the clinic?**

▢ Attending physician

▢ Fellow

▢ Nurse practitioner

▢ Physician assistant

▢ Clinic nurse

▢ Social worker

▢ Case manager

▢ Peer navigator

▢ Community Health Worker

▢ Eligibility specialist

▢ Pharmacist

▢ Research Associate

▢ Other

{Branching logic}

If other, please specify: (Text field)

1. **How long have you been employed at your clinic site?**

▢ Less than 1 month

▢ At least 1 month, but less than 6 months

▢ At least 6 months, but less than 12 months

▢ At least 1 year, but less than 3 years

▢ At least 3 years, but less than 6 years

▢ Six years or more

1. **Does your electronic medical record system have a patient portal that allows you to directly message with your patients?**

▢ Yes

{Branching logic}

If yes, how frequently do you use it to message with your patients?

▢ Never

▢ Rarely

▢ Occasionally

▢ Frequently

▢ Very frequently

▢ No

▢ Not sure

1. **Outside of the electronic medical record, are you currently using mobile health apps, tools, or websites other than PositiveLinks to enhance patient care?**

▢ I use other tools to message with patients (e.g. Whatsapp, texting on Android, iPhone, Halo)

{Branching logic}

Please specify name(s) of app/tool/website(s) used to *message* patients. (Text field)

In the past 3 months, how many times have you used this app/tool/site to *message* patients?

▢ Never

▢ Rarely

▢ Occasionally

▢ Frequently

▢ Very frequently

To what extent are you satisfied with these telemedicine services used for *messaging*?

▢ Very unsatisfied

▢ Satisfied

▢ Neutral

▢ Satisfied

▢ Very satisfied

▢ I use other tools to assist with care coordination (e.g. schedule appointments)

{Branching logic}

Please specify name(s) of app/tool/website(s) used for *care coordination*. (Text field)

In the past 3 months, how many times have you used this app/tool/site for *care coordination*?

▢ Never

▢ Rarely

▢ Occasionally

▢ Frequently

▢ Very frequently

To what extent are you satisfied with these telemedicine services used for *care coordination*?

▢ Very unsatisfied

▢ Satisfied

▢ Neutral

▢ Satisfied

▢ Very satisfied

▢ I use other tools to share lab results with patients

{Branching logic}

Please specify name(s) of app/tool/website(s) used to *share lab results?* (Text field)

In the past 3 months, how many times have you used this app/tool/site to *share lab results?*

▢ Never

▢ Rarely

▢ Occasionally

▢ Frequently

▢ Very frequently

To what extent are you satisfied with these telemedicine services used for *sharing lab results?*

▢ Very unsatisfied

▢ Satisfied

▢ Neutral

▢ Satisfied

▢ Very satisfied

▢ I use other tools to share or receive documents from patients

{Branching logic}

Please specify name(s) of app/tool/website(s) used to *share or receive documents.* (Text field)

In the past 3 months, how many times have you used this app/tool/site to *share or receive documents?*

▢ Never

▢ Rarely

▢ Occasionally

▢ Frequently

▢ Very frequently

To what extent are you satisfied with these telemedicine services used for *sharing or receiving documents?*

▢ Very unsatisfied

▢ Satisfied

▢ Neutral

▢ Satisfied

▢ Very satisfied

▢ No, I do not use other electronic tools outside of electronic medical record and PositiveLinks

**The following questions are related to your site's overall readiness to implement PositiveLinks. Please rate the**

**following items based on the scale (1-5):**

| 1 | 2 | 3 | 4 | 5 |
| --- | --- | --- | --- | --- |
| Disagree | Somewhat  Disagree | Neither Agree nor Disagree | Somewhat  Agree | Agree |

| People who work here feel confident that the organization can get people invested in implementing this change. | 1 2 3 4 5 |
| --- | --- |
| People who work here are committed to implementing this change. | 1 2 3 4 5 |
| People who work here feel confident that they can keep track of progress in implementing this change. | 1 2 3 4 5 |
| People who work here will do whatever it takes to implement this change. | 1 2 3 4 5 |
| People who work here feel confident that the organization can support people as they adjust to this change. | 1 2 3 4 5 |
| People who work here want to implement this change. | 1 2 3 4 5 |
| People who work here feel confident that they can keep the momentum going in implementing this change. | 1 2 3 4 5 |
| People who work here feel confident that they can handle the challenges that might arise in implementing this change. | 1 2 3 4 5 |
| People who work here are determined to implement this change. | 1 2 3 4 5 |
| People who work here feel confident that they can coordinate tasks so that implementation goes smoothly. | 1 2 3 4 5 |
| People who work here are motivated to implement this change. | 1 2 3 4 5 |
| People who work here feel confident that they can manage the politics of implementing this change. | 1 2 3 4 5 |
