# Supplementary material for: A Complex Digital Health Intervention to Support People With HIV: Organizational Readiness Survey Study and Preimplementation Planning for a Hybrid Effectiveness-Implementation Study
Source: J Med Internet Res. 2026 Jan 21;28:e76327. doi: 10.2196/76327 (PMC12823349; doi:10.2196/76327)
Supplement: Multimedia Appendix 3 [file jmir-v28-e76327-s003.docx]

**Multimedia Appendix 3: Provider Follow Up Survey**

**Name** (Your name will be used exclusively for the purpose of tracking characteristics of platform users. All data will be kept confidential with limited access to certain members of the study team. Summary data without identifiers will be reported to program staff.): (Text field) ***[RE-AIM - Adoption Dimension]***

**Clinic Name**: (Dropdown selection) ***[RE-AIM - Adoption Dimension]***

**What is your role in the clinic? *[RE-AIM - Adoption Dimension]***

▢ Attending physician

▢ Fellow

▢ Nurse practitioner

▢ Physician assistant

▢ Clinic nurse

▢ Social worker

▢ Case manager

▢ Peer navigator

▢ Community Health Worker

▢ Eligibility specialist

▢ Pharmacist

▢ Research Associate

▢ Other

{Branching logic}

If other, please specify: (Text field)

**Which of the following tasks do you perform to support PositiveLinks (PL) in your clinic? Please note that not all tasks listed may be applicable to you and your role. Please select N/A if the task is not applicable to you. *[RE-AIM - Implementation Dimension - Fidelity, Adaptations]***

|  | **Never** | **Occasionally** | **Frequently** | **N/A** |
| --- | --- | --- | --- | --- |
| Identify patients likely to benefit from PL and refer for consent/sign up process | ▢ | ▢ | ▢ | ▢ |
| Promote PL to other providers | ▢ | ▢ | ▢ | ▢ |
| Promote PL with patients | ▢ | ▢ | ▢ | ▢ |
| Obtain consent from patients | ▢ | ▢ | ▢ | ▢ |
| Create user accounts for PL | ▢ | ▢ | ▢ | ▢ |
| Install PL for patients and demonstrate feature functionalities | ▢ | ▢ | ▢ | ▢ |
| Troubleshoot my colleagues' technological issues with PL | ▢ | ▢ | ▢ | ▢ |
| Troubleshoot my patients' technological issues with PL | ▢ | ▢ | ▢ | ▢ |
| Assist my colleagues to navigate and use PL features | ▢ | ▢ | ▢ | ▢ |
| Perform tracking or monitoring of PL activities | ▢ | ▢ | ▢ | ▢ |
| Participate in discussions to improve how my site implements PL | ▢ | ▢ | ▢ | ▢ |
| Other | ▢ | ▢ | ▢ | ▢ |

**We are interested in understanding how you use the messaging feature of the PositiveLinks (PL) platform to communicate with patients. If your site did not use the messaging feature please select N/A, if you did use the messaging feature at your site please detail the reason for the communication use and its frequency.**

**In my role at my clinic site, I typically use the PL messaging features to communicate with my patients to: *[RE-AIM - Implementation Dimension - Fidelity, Adaptations]***

|  | **Never** | **Occasionally** | **Frequently** | **N/A** |
| --- | --- | --- | --- | --- |
| Coordinate appointment scheduling | ▢ | ▢ | ▢ | ▢ |
| Facilitate medication refills | ▢ | ▢ | ▢ | ▢ |
| Assist with patient insurance applications or related eligibility  concerns | ▢ | ▢ | ▢ | ▢ |
| Share lab results | ▢ | ▢ | ▢ | ▢ |
| Discuss HIV diagnosis, comorbidities and/or treatment | ▢ | ▢ | ▢ | ▢ |
| Address non-medical concerns related to care (e.g. housing, transportation) | ▢ | ▢ | ▢ | ▢ |
| Provide or refer to specific educational resources available on PositiveLinks | ▢ | ▢ | ▢ | ▢ |
| Other | ▢ | ▢ | ▢ | ▢ |

**We are interested in understanding how you use PositiveLinks (PL) to communicate with other providers at your clinic site. *[RE-AIM - Implementation Dimension - Fidelity, Adaptations]***

|  | **Never** | **Occasionally** | **Frequently** | **N/A** |
| --- | --- | --- | --- | --- |
| To what extent do you use PL to communicate with other providers through the messaging feature? | ▢ | ▢ | ▢ | ▢ |

**We are interested in understanding which features of the PositiveLinks (PL) platform you use the most, and which you do not use at all. For each feature, we've listed the typical way each feature is used, but please check "I use it in another way" if you use this feature differently, and describe how you use it. *[RE-AIM - Implementation Dimension - Fidelity, Adaptations]***

**In my role at the clinic, I use the:**

| Check-ins to review/track patient responses to daily  check-ins (mood, stress, medications) | **Never** | **Occasionally** | **Frequently** | **I use it in another way** |
| --- | --- | --- | --- | --- |
|  | ▢ | ▢ | ▢ | ▢  {Branching logic}  If another usage, please specify: (Text field) |
| Lab results to review/track changes in patient lab results over time | ▢ | ▢ | ▢ | ▢  {Branching logic}  If another usage, please specify: (Text field) |
| Document upload to obtain documents uploaded by  patient | ▢ | ▢ | ▢ | ▢  {Branching logic}  If another usage, please specify: (Text field) |
| Appointments to update patient appointment  information within PL | ▢ | ▢ | ▢ | ▢  {Branching logic}  If another usage, please specify: (Text field) |
| Contacts to obtain contact information for providers  outside of your site listed in the PL directory | ▢ | ▢ | ▢ | ▢  {Branching logic}  If another usage, please specify: (Text field) |

| **How satisfied are you with these features?** | **Very unsatisfied** | **Unsatisfied** | **Neutral** | **Satisfied** | **Very satisfied** |
| --- | --- | --- | --- | --- | --- |
|  | ▢ | ▢ | ▢ | ▢ | ▢ |
| **To what extent do you think the PositiveLinks portal meets your needs in providing patient care?** | **Not at all** | **A little** | **Somewhat** | **Very** | **Extremely** |
|  | ▢ | ▢ | ▢ | ▢ | ▢ |
| **To what extent do you think the PositiveLinks smartphone app meets your needs in providing patient care?** | **Not at all** | **A little** | **Somewhat** | **Very** | **Extremely** |
|  | ▢ | ▢ | ▢ | ▢ | ▢ |

**If PositiveLinks was available in your clinic, how likely is it that you would continue to use it outside of the study? *[RE-AIM - Maintenance Dimension]***

|  | **Not at all** | **A little** | **Somewhat** | **Very** | **Extremely** |
| --- | --- | --- | --- | --- | --- |
|  | ▢ | ▢ | ▢ | ▢ | ▢ |

**Why or why not? Please specify:** (Text field)

**To what extent is PositiveLinks compatible with the following: *[CFIR - Inner Setting: Compatibility]***

|  | **Not at all** | **A little bit** | **Somewhat** | **Quite a bit** | **Very much** |
| --- | --- | --- | --- | --- | --- |
| The clinic workflow to take care of patients | ▢ | ▢ | ▢ | ▢ | ▢ |
| The clinic's goals to improve medication adherence | ▢ | ▢ | ▢ | ▢ | ▢ |
| The clinic's goals to provide patient centered care | ▢ | ▢ | ▢ | ▢ | ▢ |
| The clinic's goals to connect with patients | ▢ | ▢ | ▢ | ▢ | ▢ |
| The staff's efforts to engage patients in care | ▢ | ▢ | ▢ | ▢ | ▢ |
| The staff's efforts to overcome communication barriers | ▢ | ▢ | ▢ | ▢ | ▢ |

**To what extent does PositiveLinks provide a way to improve patients: *[CFIR - Outer Setting: Patient Needs & Resources, Characteristics of Individuals: Knowledge & Beliefs]***

|  | **Not at all** | **A little bit** | **Somewhat** | **Quite a bit** | **Very much** |
| --- | --- | --- | --- | --- | --- |
| Communication with their providers | ▢ | ▢ | ▢ | ▢ | ▢ |
| Access to information | ▢ | ▢ | ▢ | ▢ | ▢ |
| Attending in-person visits with their provider | ▢ | ▢ | ▢ | ▢ | ▢ |
| Having a positive connection with their provider | ▢ | ▢ | ▢ | ▢ | ▢ |
| Having a positive connection with their clinic | ▢ | ▢ | ▢ | ▢ | ▢ |
| Engagement with the care setting and process of care | ▢ | ▢ | ▢ | ▢ | ▢ |
| Engagement with the process of living well with HIV | ▢ | ▢ | ▢ | ▢ | ▢ |
| Social support | ▢ | ▢ | ▢ | ▢ | ▢ |
| Access to lab results | ▢ | ▢ | ▢ | ▢ | ▢ |
| Mental health | ▢ | ▢ | ▢ | ▢ | ▢ |

**To what extent would you rate the following potential clinic or provider barriers to using PositiveLinks?**

|  |  | **Not at all a barrier** | **Minor barrier** | **Major barrier** |
| --- | --- | --- | --- | --- |
| ***[CFIR - Inner Setting: Compatibility]*** | Changing communication with clients from the Electronic Health Record to PL | ▢ | ▢ | ▢ |
| ***[CFIR - Inner Setting: Compatibility]*** | Receiving messages from clients outside of clinic | ▢ | ▢ | ▢ |
| ***[CFIR - Outer Setting: External Policy & Incentives]*** | Concerns about information security and privacy | ▢ | ▢ | ▢ |
| ***[CFIR – Implementation Process: Engaging***  ***Key Stakeholders]*** | Staff buy-in to the PL implementation | ▢ | ▢ | ▢ |
| ***[CFIR - Implementation Process: Engaging***  ***Key Stakeholders]*** | Leadership buy-in to PL | ▢ | ▢ | ▢ |
| ***[CFIR - Implementation Process: Planning]*** | Disorganized roll-out processes of PL in my clinic | ▢ | ▢ | ▢ |
| ***[CFIR - Inner Setting: Compatibility]*** | Provider preference for current systems to communicate with patients | ▢ | ▢ | ▢ |
| ***[CFIR - Inner Setting: Compatibility]*** | Provider preference for current systems and practice to care for patients | ▢ | ▢ | ▢ |

**To what extent would you rate the following potential patient barriers to using PositiveLinks?**

|  |  | **Not at all a barrier** | **Minor barrier** | **Major barrier** |
| --- | --- | --- | --- | --- |
| ***[CFIR - Inner Setting: Compatibility]*** | Patients access to lab results prior to discussion with clinic | ▢ | ▢ | ▢ |
| ***[CFIR - Outer Setting: External Policy & Incentives]*** | Patient concerns about information security and privacy | ▢ | ▢ | ▢ |
| ***[CFIR – Innovation Characteristics: Adaptability,***  ***Complexity]*** | Patient difficulty in using the phone | ▢ | ▢ | ▢ |
| ***[CFIR - Innovation Characteristics: Adaptability]*** | Patient lack of interest in PositiveLinks | ▢ | ▢ | ▢ |
| ***[CFIR - Inner Setting: Compatibility]*** | Patient preference for current systems to communicate with providers | ▢ | ▢ | ▢ |

|  |  |  |  |
| --- | --- | --- | --- |
